# Bowel necrosis in patient with severe case of COVID-19: a case report

**DOI:** 10.1186/s12893-021-01104-7

**Published:** 2021-02-22

**Authors:** Daniel Ardian Soeselo, Wirawan Hambali, Sandy Theresia

**Affiliations:** 1 Department of Surgery, School of Medicine and Health Science, Atma Jaya Catholic University of Indonesia, Pluit Raya No. 2, North Jakarta, 14440 Jakarta Indonesia; 2 Department of Surgery, Pondok Indah Puri Indah Hospital, Puri Indah Raya S-2, West Jakarta, Jakarta 11610 Indonesia; 3 Department of Internal Medicine, Pondok Indah Puri Indah Hospital, Puri Indah Raya S-2, West Jakarta, 11610 Jakarta Indonesia

**Keywords:** COVID-19, General surgery, Bowel necrosis

## Abstract

**Background:**

In patients who are critically ill with COVID-19, multiple extrapulmonary manifestations of the disease have been observed, including gastrointestinal manifestations.

**Case presentation:**

We present a case of a 65 year old man with severe COVID-19 pneumonia that developed hypercoagulation and peritonitis. Emergent laparotomy was performed and we found bowel necrosis in two sites.

**Conclusions:**

Although rare, the presentation of COVID-19 with bowel necrosis requires emergency treatments, and it has high mortality rate.

## Background

The novel Coronavirus disease (COVID-19) is a disease caused by the severe acute respiratory syndrome coronavirus 2 (SARS-CoV-2), it has spread worldwide and has become a pandemic. COVID-19 is currently causing more than 900.000 deaths. Indonesia is one of the countries with the highest number of registered cases and deaths [1].

The Coronavirus belongs to a family of RNA viruses that can cause respiratory infection with varying symptoms, which usually includes cough, fever, fatigue, headache and myalgia after 2–14 days of exposure. This virus enters the human cells through the angiotensin-converting enzyme-2 (ACE-2) receptor, which is also expressed in the gastrointestinal tract (GIT) epithelium. GIT involvement can be characterized by abdominal pain, hyporexia, nausea, and vomiting with a variable incidence ranging from 5 to 50% of cases [2, 3].

In addition, COVID-19 may also predispose to venous and arterial thromboembolic diseases due to excessive inflammation, hypoxia and diffuse intravascular coagulation through the triad of hypercoagulation, blood stasis, and endothelial injury. This occurrence of vascular thickening and thromboembolic resulting in increased hypoxemia with predictive value of adverse outcomes associated with d-dimer levels [2, 4].

In this case report, we describe a patient with COVID-19 who developed a bowel necrosis that required emergent surgical treatment.

## Case presentation

**Fig. 1:**
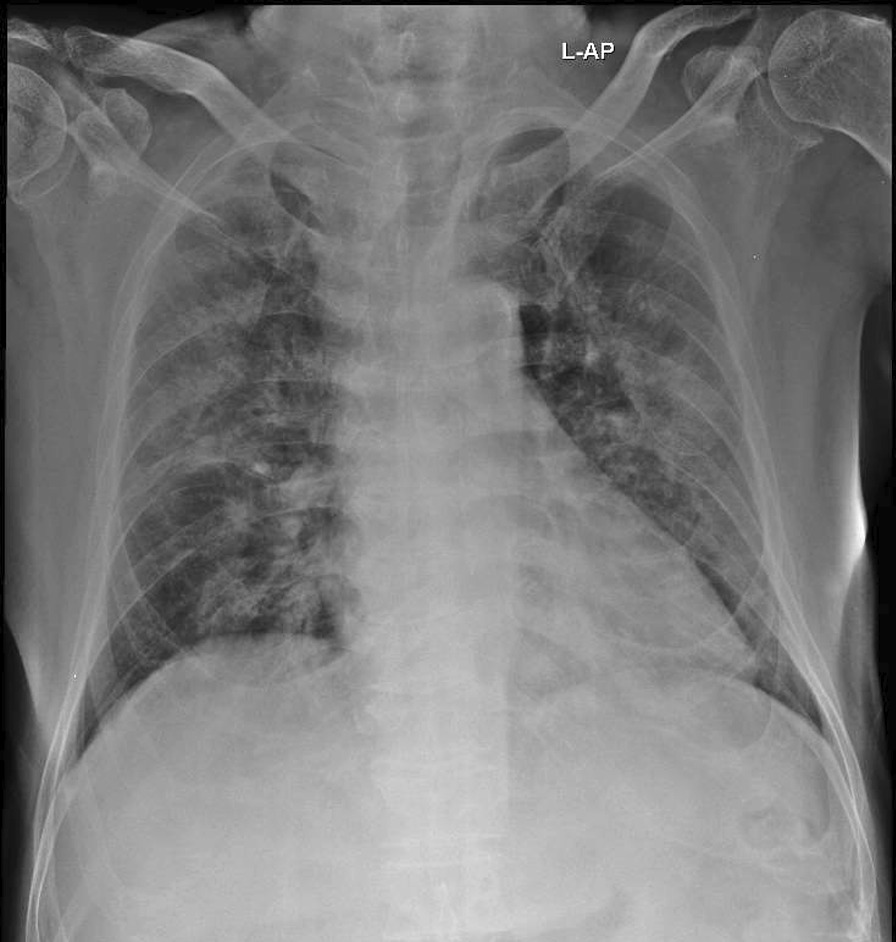
Chest X-ray showing bilateral pneumonia

A 65-year-old male patient with past medical history for diabetes mellitus was brought to the hospital for having 7 days of fever and cough, and recent onset of dyspnea. The patient was given 15 lpm of oxygen with non-rebreathing mask, and his oxygen saturation improved from 85 to 90%. Laboratory tests were as follows: haemoglobin (Hb) 16.2 g/dL; leucocyte 9000/μL; lymphocyte 3%; thrombocytes 222,000/μL; CRP 0,2 mg/dL; d-dimer 0.78 mg/L; HbA1c 8.0%. His chest x-ray showed severe bilateral pneumonia (Fig. 1) and his real-time PCR on nasopharyngeal and oropharyngeal swabs were tested positive for SARS-COV-2. The patient was diagnosed with COVID-19 with severe case and received COVID-19 regimen.

**Fig. 2:**
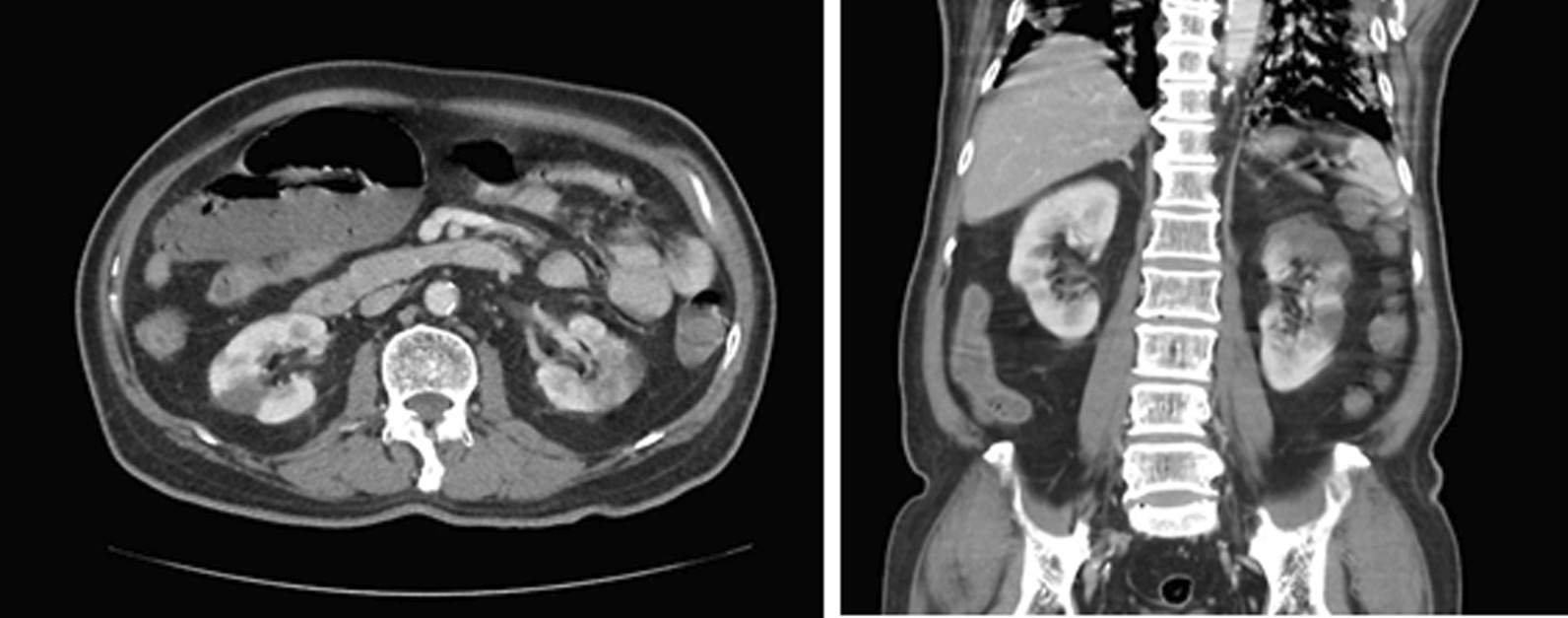
Abdomen CT scan showing bilateral renal infarction

After 7 days of hospitalized, the patient suddenly complained of epigastric pain. His respiratory rate was increased, oxygen saturation was 88–90% with 15 lpm of oxygen, and there was tenderness in the epigastric. Laboratory results showed an increase in leucocyte 15.100/μL; D-dimer 14.04 mg/L and Albumin 1.4 g/dL. A contrast abdominal CT scan was performed, infarction was found in both kidneys and no abnormalities in other abdominopelvic organ (Fig. 2). Abdominal pain was suspected due to mesentery ischemia caused by hypercoagulation because ischemia was seen on the kidneys, whereas renal arteries are bigger than mesenteric arteries. Anticoagulant therapy with low-molecular weight heparin 25,000 IU/day were administered; with the addition of tramadol 100 mg/day to reduced the pain and third-generation of cephalosporin antibiotic as bowel decontamination. On the next day, the patient felt less abdominal pain, d-dimer decreased to 8.54/μL and the medications were continued.

**Fig. 3:**
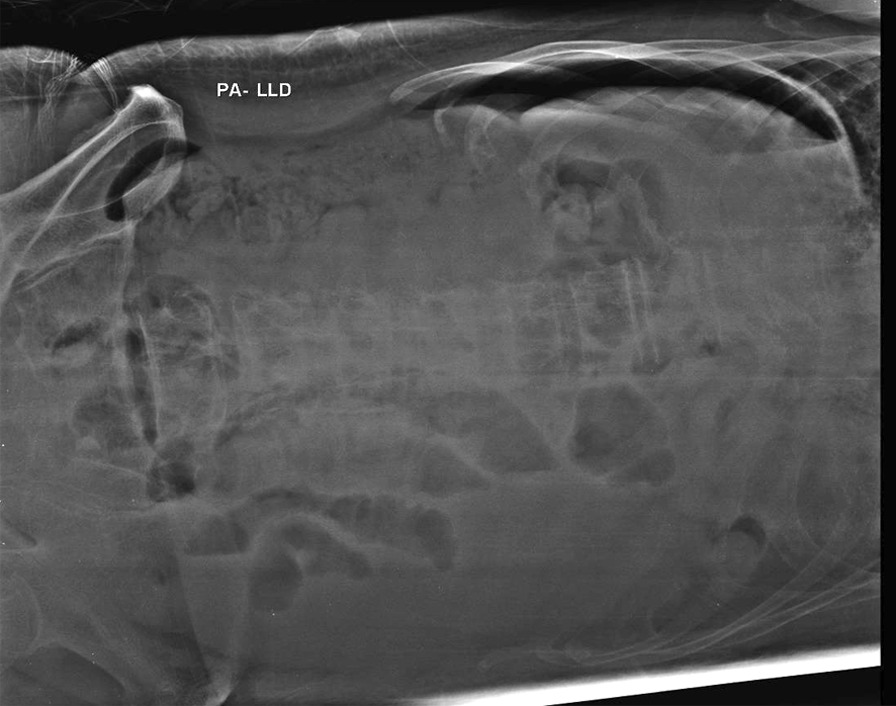
Abdomen X-ray in left lateral decubitus showing free air in the abdominal cavity

Three days after his epigastric pain, the patient complained of had a worsened abdominal pain with marked distension, inability to pass gas or defecate. On physical examination, signs of peritoneal irritation were found. His leucocyte increased to 33.100/μL; aPTT 71.4 s (control aPTT 24.1 s). Abdominal X-ray showed free air in the abdominal cavity and distension of the large bowel (Fig. 3). We diagnosed it as intestinal perforation in COVID-19.

**Fig. 4:**
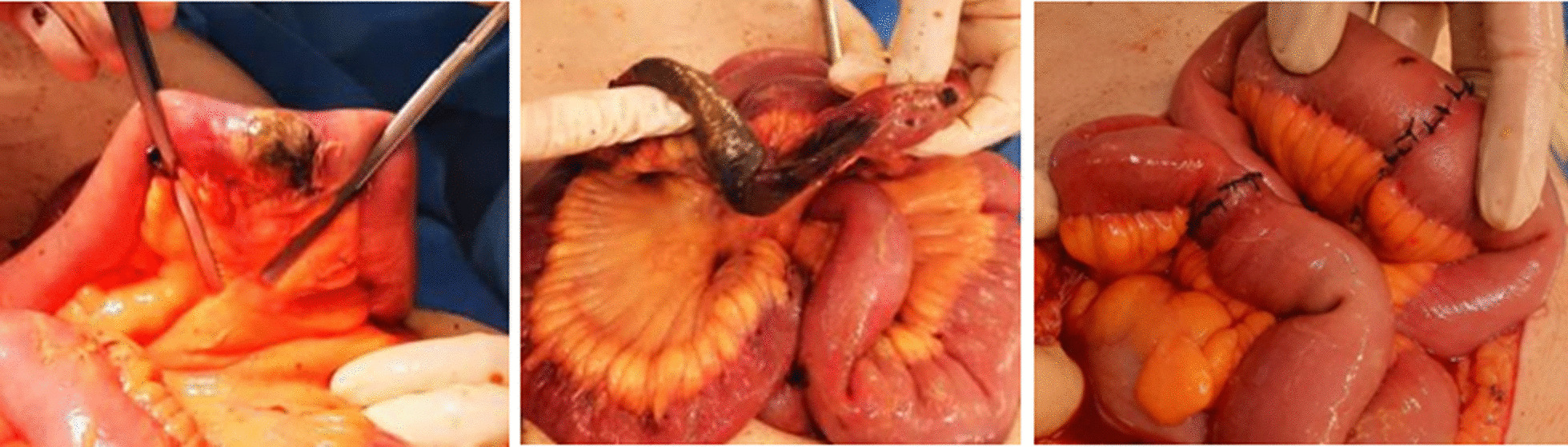
(Left) 5 cm necrosis 170 cm from Treitz ligament, (Middle) 20 cm necrosis one meter from the first one, and (Right) after bowel resection and end-to-end anastomose performed

**Fig. 5:**
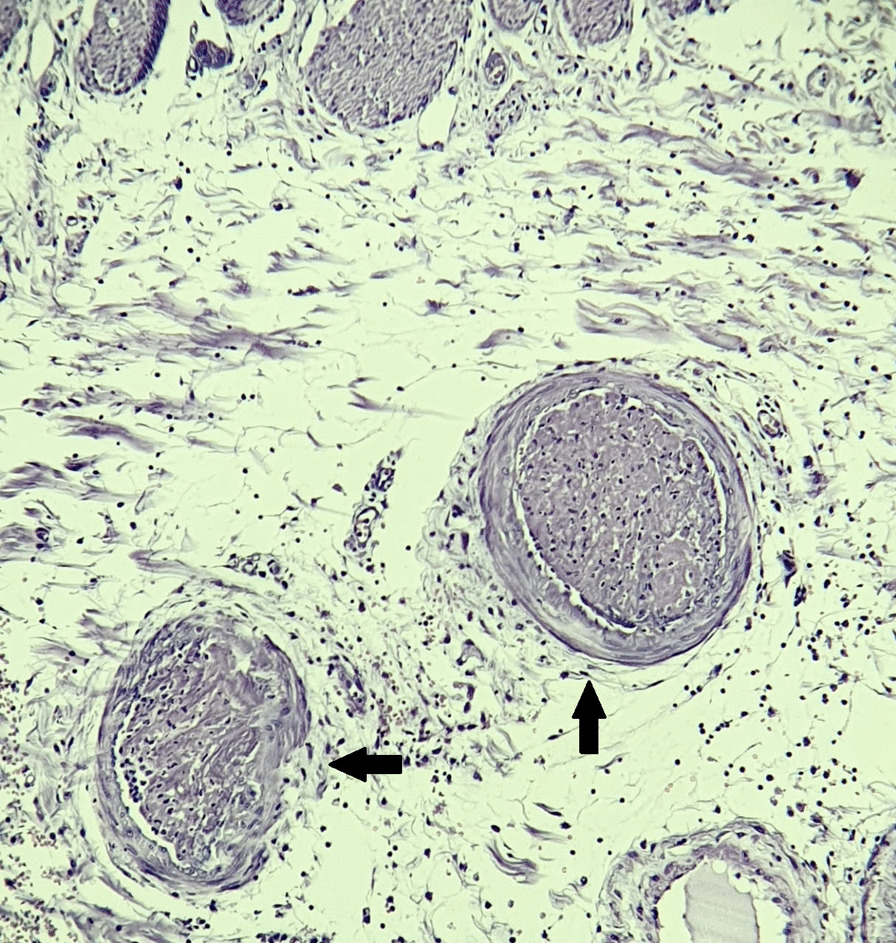
Histology showing multiple microthrombus in submucosa of small bowel tissue

The patient underwent an emergent laparotomy with midline incision. The surgery was performed under general anaesthesia. After the peritoneum was incised, air came out of the peritoneal cavity and the bowel was soaked in brownish liquid and reddish black feces. We explored it and obtained about 200 cc of brownish liquid. During the surgery, we found necrosis in two locations: first, we found a round necrosis, 3 cm in diameter, yellowish black in antimesenterial site. In the second location, 20 cm whole intestinal necrosis was found at 1 m from the first site (Fig. 4). We did bowel resection at each site of necrosis and end-to-end anastomoses by using manual suturing (Fig. 4). The surgery duration was approximately 2 h and at the end of the surgery, intra-abdominal drain was placed to help further evaluation of re-perforation occurrence. The histology showed a small bowel tissue with multiple microthrombus in submucosa, extensive haemorrhagic infarction and perforation (Fig. 5).

Right after surgery, the patient was admitted to the ICU and was put on ventilator, with anticoagulant and other medications continuing. The patient had stable vital signs with 85–93% oxygen saturation on ventilator, but never regained consciousness. On the fourth day, melena occurred and blood laboratory result showed Hb 8 mg/dL, ureum 1.4 mg/dL, and creatinin 42 mg/dL. The patient was given packed red cell (PRC) and fresh frozen plasma (FFP), 500 cc each. We did not find any sign of intestinal paralytic, discharge from the drain and there were no signs of re-perforation. Six day after surgery, the patient’s condition got worse with unstable vital signs and progressive decrease of oxygen saturation. Unfortunately, the patient passed away in the ICU. We did not perform autopsy because the patient’s family refused.

## Discussion and conclusions

Several researchers have reported the link between COVID-19 and generalized organ damage, including organs in the abdominopelvic. This association is thought to be because SARS-CoV-2 binds to the angiotensin-converting enzyme 2 (ACE-2) receptor found in alveolar epithelial cells in the lung, and these receptors are also detected in gastrointestinal epithelial cells resulting in infection and local viral replication, and increase cytotoxic effect [4, 5]. In addition, some authors also think that organ damage in some COVID-19 patients may be caused by severe systemic inflammation caused by upregulation of cellular and natural immunity. SARS-CoV-2 infection triggers the activation of T lymphocytes and the inflammatory signaling pathway which ultimately results in the release of multiple proinflammatory cytokine, such as granulocyte–macrophage colony-stimulating factor (GM-CSF), interleukin (IL)-2, IL-6, IL-7, IL-10, and tumor necrosis factor-α (TNF-α) [6]. This cytokine cascade can eventually result in extensive cell damage, necrosis, and injury to multiple organs and may partly explain the different multisystem symptoms in patients with confirmed viral infections, including gastrointestinal necrosis [6, 7].

Intestinal necrosis is a late stage discovery characterized by cell death due to reduced blood flow to the digestive tract. This serious condition is often fatal and can lead to vascular occlusion, colitis, obstruction, or infection. In adults, the most common cause of intestinal necrosis is acute mesenteric occlusion, and, less commonly are, perforation, chronic ischemia, inflammatory disease and other mechanical disorders [8, 9]. In our case, the bowel necrosis we found had patent blood vessels and did not involve mesenteric necrosis, therefore, we think that it might be due to microvascular thrombosis and the inflammation associated with hypercoagulability in this patient. A case reported by Gartland et al. also found a similar case in which a patient with COVID-19 had complaints of abdominal pain and developed intestinal necrosis [10]. Several studies have recently investigated the association between COVID-19 and hypercoagulability which is usually characterized by high d-dimer [11, 12]. Some patients may show prolonged thrombin time and prothrombin time, and shortened aPTT. Several other studies have shown aPTT prolongation in patients with confirmed COVID-19 [12].

Bowel necrosis without adequate intervention and proper surgical management will lead to near hundred percent of mortality. The operative mortality rate for acute mesenteric ischemia has been reported to be 47%. Patients who survive the initial event have a high probability of postoperative complications [13, 14]. Sepsis can cause hypotension and end-organ damage, especially kidney and liver failure. For those who survive the early intervention, mortality continues to increase from comorbidities [14].

Although rare, the emergent surgery for bowel necrosis and COVID-19 infection presents unique challenges for clinicians.

## Data Availability

Not applicable.

## Abbreviations

ACE-2: Angiotensin-converting enzyme-2
aPTT: Activated partial thromboplastin time
COVID-19: Coronavirus disease 2019
CRP: C-reactive protein
GM-CSF: Granulocyte–macrophage colony-stimulating factor
ICU: Intensive Care Unit
IL: Interleukin
PCR: Polymerase chain reaction
SARS-CoV-2: Severe acute respiratory syndrome coronavirus 2
TNF-α: Tumor necrosis factor alpha
